# B-type natriuretic peptide for assessment of haemodynamically significant patent ductus arteriosus in premature infants

**DOI:** 10.1111/apa.12273

**Published:** 2013-05-10

**Authors:** Kenji Mine, Atsushi Ohashi, Shoji Tsuji, Jun-ichi Nakashima, Masato Hirabayashi, Kazunari Kaneko

**Affiliations:** Department of Pediatrics, Kansai Medical UniversityOsaka, Japan

**Keywords:** B-type natriuretic peptide, Haemodynamically significant patent ductus arteriosus, Indomethacin, Premature infants, Surgical ligation

## Abstract

**Aim**: Haemodynamically significant patent ductus arteriosus (hsPDA) is frequently observed in premature infants. This study was conducted to explore whether the blood BNP can be a valuable biomarker to assess the necessity of treatment for hsPDA in premature infants.

**Methods**: Serial measurements of the blood BNP were performed during the first 5 days of life in premature infants with hsPDA (Group I) and those without hsPDA (Group N). The definition of the hsPDA was the PDA requiring treatment, such as indomethacin administration and/or surgical ligation.

**Results**: Forty-six subjects were enrolled. Compared with Group N, Group I showed significantly higher level of blood BNP at postnatal 24–96 h and demonstrated the peak value at postnatal 24–48 h. With the ROC curve using the data at postnatal 24–48 h in Group I, we deduced the predictive value of 250 pg/mL of blood BNP for indomethacin treatment. Similarly, with the ROC curve using the maximal value of blood BNP within the first 5 days of life, the predictive value of 2000 pg/mL for surgical ligation was deduced.

**Conclusions**: Blood BNP during early postnatal period can be a useful biomarker to assess the necessity of treatment for hsPDA in premature infants.

## Introduction

Haemodynamically significant patent ductus arteriosus (hsPDA) is observed in more than 30% of premature infants with gestational ages of <32 weeks 1. When the ductus arteriosus (DA) is open after birth, a left-to-right shunt occurs via DA. As a result, the systemic blood flow is decreased, while the pulmonary blood flow is increased. The decrease in the systemic blood flow reduces the intestinal and renal blood flow, and the increase in the pulmonary blood flow may set up congestive heart failure, cause pulmonary haemorrhage, lead to intracranial haemorrhage and exacerbate chronic lung diseases 2. Therefore, hsPDA is considered to be associated with various comorbid illnesses in premature infants.

In general, the clinical diagnosis of hsPDA is made by echocardiography once hsPDA is suspected based on physical observations, such as a heart murmur, increased heart rate or widened pulse pressure. For the treatment of hsPDA, indomethacin, a prostaglandin synthesis inhibitor, is effective and yields DA closure in 70–80% of premature infants 3. While it is considered that treatment in the early postnatal period results in a high closure rate 4,5, administering indomethacin to premature infants may also induce severe side effects including renal failure, gastrointestinal tract perforation and hypoglycaemia. Some have postulated that an echocardiography can be used not only to make a diagnosis but also to assess the timing and amount of indomethacin treatment, that is, an enlarged DA diameter 6, increased pulmonary arterial blood flow 7,8, disruption of the diastolic blood flow in the superior mesenteric artery 6 and increased ratio between the left atrium and the internal diameter of the aorta 6,8. However, there is no uniform therapeutic strategy in regard to the timing or amount of indomethacin administration as the treatment for hsPDA in premature infants.

B-type natriuretic peptide (BNP), which has vasodilating and diuretic effects and plays an important role in regulating the body fluid volume and blood pressure 9, is specifically secreted from the cardiac ventricle into the circulation in response to increased cardiac stress. Accordingly, BNP has been used as a biomarker for the diagnosis and assessing the response to the treatment of chronic heart failure in adults 9. Even in paediatric patients, it has been also postulated that serum levels of BNP help us to assess the children with congenital heart diseases 10. Furthermore, it has been recently reported that it could be a useful biomarker for the severity of hsPDA in premature infants 11–13.

Taken together, we explored whether an early postnatal blood BNP level can be a useful biomarker to assess the necessity of therapeutic interventions for hsPDA in premature infants.

## Subjects and Methods

**Figure 1:**
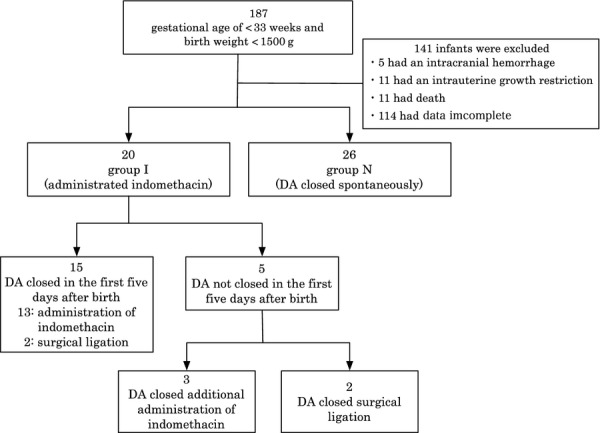
Flow chart of participant entry. DA, ductus arteriosus.

The subjects included 46 premature infants admitted to the neonatal intensive care unit (NICU) of the Kansai Medical University Hospital between October 2007 and July 2010, with a gestational age of <33 weeks and a birth weight of <1500 g. We excluded the subjects with chromosomal abnormalities, congenital heart failure except those due to patent ductus arteriosus, intrauterine growth restriction or intracranial haemorrhage from the study because of the possibility that these conditions influence the blood level of BNP Fig. 1.

We divided the enrolled subjects into two groups and measured blood BNP levels for first 5 days since birth: the 20 patients with hsPDA, who needed indomethacin treatment and/or surgical ligation, were categorized as intervention group (Group I), while the remaining 26 patients without hsPDA, who did not require medical interventions as their DA closed spontaneously, were categorized as noninterventional group (Group N). The diagnosis of hsPDA was made by the echocardiography using a Xario instrument (Toshiba Medical Systems, Tokyo, Japan) performed on the time of admission and every 12 h by the trained paediatricians who were blinded to the results of the blood BNP level.

The subjects were diagnosed as having hsPDA and were treated with indomethacin when at least one of the followings was observed: (i) the end-diastolic blood flow velocity of the left pulmonary artery was above 30–40 cm/sec 7, (ii) the diastolic blood flow of the anterior cerebral artery was interrupted 6 or (iii) the diastolic blood flow of the superior mesenteric artery was interrupted 6. In addition, when the DA did not close despite the repetitive administration of indomethacin (maximum four times [median 1.5 times], maximum dose 0.7 mg/kg [median 0.2 mg/kg]), and there were no changes in the echocardiographic findings, we performed surgical ligation on 3- to 17-day-old premature infants (median 9.5 days of age).

The blood BNP level was measured once a day using whole blood at postnatal 0–24, 24–48, 48–72, 72–96 and 120–144 h in all subjects enrolled in the study. Accordingly, there was a lapse of time in hours as long as 12 h between the echocardiographic examination and blood sampling for BNP measurement.

For each subject, a 0.1 mL whole blood sample was collected as a part of the routine blood examinations via the catheter coated with 2K-EDTA inserted into an artery for pressure monitoring. After obtaining the blood sample, the BNP level was immediately measured by a BNP rapid assay kit (Shionospot BNP®; Shionogi Pharmaceutical Co., Osaka, Japan), with a dedicated instruments: this is based on fluorescence-labelled immunochromatography, and we followed the manufacturer’s instructions regarding its operation. According to the manufacturer’s specifications, measuring range of Shionospot BNP® was 6–2000 pg/mL, and its coefficient of variation and accuracy using the quality control are <15% and within 30%, respectively.

The subjects were retrospectively analysed in the followings: (i) the blood BNP levels at postnatal 0–24, 24–48, 48–72, 72–96 and 120–144 h were compared between Group I and Group N; (ii) in order to set a BNP cut-off value for indomethacin treatment, a receiver operating characteristic (ROC) curve was drawn using the BNP data before the treatment with indomethacin at postnatal 24–48 h of the subjects in Group I. Using the generated cut-off value, an odds ratio for the necessity of indomethacin was also calculated; (iii) in order to set a BNP cut-off value for surgical ligation, a ROC curve was drawn using the maximal blood value of BNP within the first 5 days of life in Group I. Using the generated cut-off value, an odds ratio for the surgical ligation was calculated as well.

This study was approved by the Kansai Medical University hospital ethical committee (authorized number: H090403). The informed consent was also obtained from the patients’ parents.

### Statistical analysis

All of the statistical analyses were performed on a personal computer with the statistical package for social sciences (Excel Statistics 2010; SSRI, Tokyo, Japan) for Windows software package.

The numerical data were determined as a nonparametric distribution, and the representative value was indicated as a median value with a range. For statistical comparisons to compare the numerical data in two groups, a Mann–Whitney U-test was used. To compare the categorical data in the two groups, a Fisher’s exact test was used. A statistically significant difference was defined as a value of p < 0.05 (two-tailed) in each test.

## Results

### Clinical characteristics of the patients

A flow chart regarding an eligibility into the current study was shown in Figure 1. There were no significant differences between Group I and Group N in terms of their gestational age at birth, birth weight, the ratio of male and female infants, mode of delivery, Apgar scores at 1 and 5 min, the use of catecholamine or the use of antenatal steroids Table 1. As mentioned before, all cases who received ventilatory support, and those of pulmonary hypertension, sepsis and congenital heart disease were excluded because of the possible increased level of BNP not due to the presence of hsPDA.

**Table 1:** Clinical characteristic of premature infants with and without hsPDA

	Premature infants with hsPDA (Group I, n = 20)	Premature infants without hsPDA (Group N, n = 26)	p-Value
Gestational age (weeks): median (interquartile range)	28.0 (27.0–29.2)	28.1 (25.5–29.2)	0.991
Weight (g): median (interquartile range)	960 (735–1137)	950 (799–1181)	0.765
Male gender, n (%)	13 (65)	13 (50)	0.309
Caesarean section, n (%)	18 (90)	24 (92)	0.801
Apgar score at 1 min	5 (3–6)	5 (3–6)	0.991
Apgar score at 5 min	7 (5–8)	8 (6–9)	0.155
Catecholamine, n (%)	6 (30)	6 (23)	0.596
Antenatal steroid, n (%)	9 (45)	8 (31)	0.322

hsPDA, haemodynamically significant patent ductus arteriosus.

### Blood BNP level in premature infants during the early postnatal period

**Figure 2:**
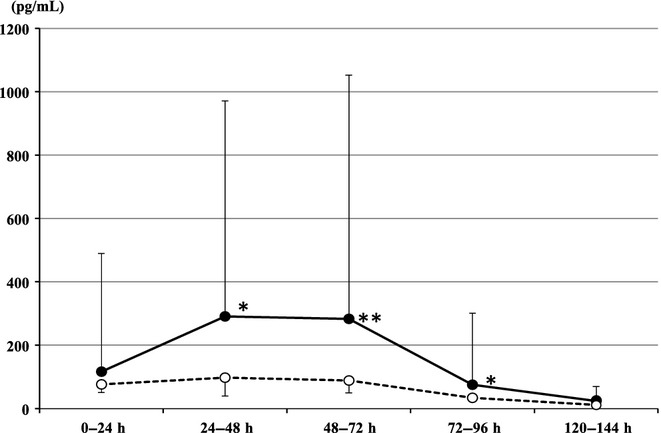
Blood BNP value in Group I (treatment) and Group N (no treatment) during the first 5 days of life. Group I (closed circles) showed significantly higher level of blood BNP at postnatal 24–48, 48–72 and 72–96 h compared with those of Group N (open circles). The circles represent the median value and the vertical lines extend from the circles to the 75th (Group I) and 25th (Group N) percentiles. *p < 0.05, **p < 0.01.

As shown in Figure 2, there were no significant differences in the BNP levels between Group I and Group N at postnatal 0–24 h of age: Group I, median 116.7 (interquartile range: 55.5–373.2) pg/mL; Group N, 76.7 (26.2–374.8) pg/mL, p = 0.595. In contrast, it was significantly higher in Group I than in Group N at postnatal 24–48 h of age: Group I, 291.1 (88.4–679.4) pg/mL; Group N, 98.3 (58.9–183.1) pg/mL, p = 0.042. In a similar fashion, in infants of postnatal 48–72 and 72–96 h, it was significantly higher in Group I than in Group N (postnatal 48–72 h of age: Group I, 283.4 (123.1–226.2) pg/mL; Group N, 88.4 (38.6–191.4) pg/mL, p = 0.0014; postnatal 72–96 h of age: Group I, 75.3 (33.4–226.2) pg/mL; Group N, 34.4 (13.9–64.9) pg/mL, p = 0.028. At the age of postnatal 120–144 h, there was no difference between Group I and Group N: Group I, 23.9 (5.9–46.2) pg/mL; Group N, 11.9 (5.9–43.9) pg/mL, p = 0.684 Fig. 2.

### Daily changes of the blood BNP in premature infants with hsPDA

As shown in Figure 2, premature infants with hsPDA in Group I revealed significantly higher values of blood BNP at 24–48 and 48–72 h of ages than those at postnatal 0–24 h of age (p = 0.033, p = 0.04, respectively), whereas there were no significant differences among the infants of postnatal 0–24, 24–48 and 48–72 h of ages in Group N.

### Days to the closure of DA in premature infants

The median days of DA closure since birth was 2 days in Group N, whereas it was significantly extended to 4 days in Group I except the subjects receiving a surgical ligation (p < 0.01).

### Association of blood BNP level with indomethacin treatment

**Figure 3:**
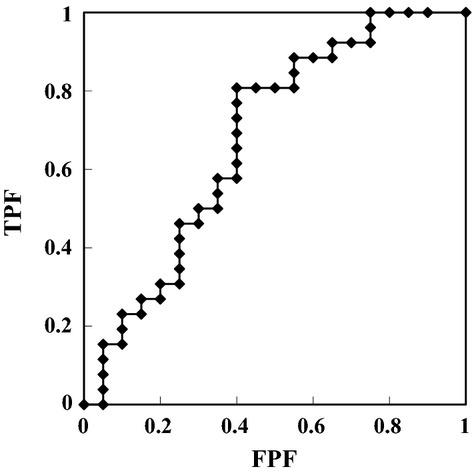
The ROC curve of the blood BNP value in premature infants with hsPDA predicting indomethacin treatment. A cut-off value of 250 pg/mL was calculated for predicting the indomethacin treatment for hsPDA according to the criteria adopted in our NICU based on the ROC curve (ROC, receiver operating characteristic; BNP, B-type natriuretic peptide; hsPDA, haemodynamically significant patent ductus arteriosus).

Indomethacin was administered at either early postnatal period (from 6 to 72 h) in 17 patients or later period (day 4 to 7) in three patients in Group I. In order to identify a cut-off value of blood BNP for the necessity of indomethacin, we drew a ROC curve using the blood BNP level at postnatal 24–48 h in patients with hsPDA (Group I), As a result, a cut-off value of 250 pg/mL was calculated based on the ROC curve Fig.3: area under the curve (AUC), 0.68; true-positive fraction (TPF), 0.80; false-positive fraction (FPF) 0.40. Based on this finding, when the cut-off value of the BNP level for predicting the necessity of indomethacin for hsPDA was set to 250 pg/mL, it generated an odds ratio as high as 5.0 (95% CI, 1.4 to 17.9, p = 0.016); indomethacin administration rate was 66.7%, while DA spontaneous closure rate was 71.4%. Table 2.

**Table 2:** Best predictive value of the blood BNP of the indomethacin treatment for hsPDA according to the criteria adopted in our NICU

BNP concentration	Group I	Group N
≥250 pg/mL	12	6
<250 pg/mL	8	20

BNP, B-type natriuretic peptide; hsPDA, haemodynamically significant patent ductus arteriosus; NICU, neonatal intensive care unit.

When the cut-off value of the BNP for hsPDA was set to 250 pg/mL for predicting the necessity of indomethacin, it generated an odds ratio as high as 5.0 (95% CI, 1.4 to 17.9, p = 0.016); indomethacin administration rate was 66.7%, while DA spontaneous closure rate was 71.4%.

### Association of blood BNP level with surgical ligation

**Figure 4:**
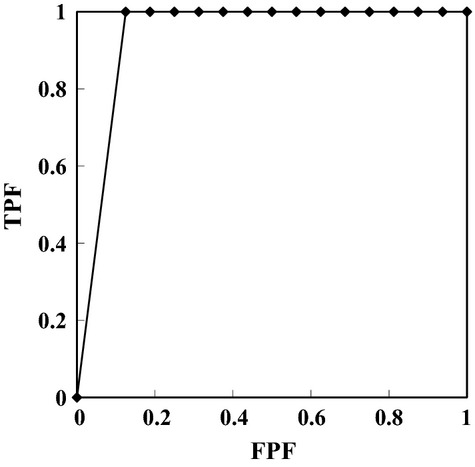
The ROC curve of the maximal blood BNP value within the first 5 days of life in premature infants with hsPDA undergoing surgical ligation. A cut-off value of 2000 pg/mL was calculated for predicting the surgical ligation for hsPDA according to the criteria adopted in our NICU based on the ROC curve (ROC, receiver operating characteristic; BNP, B-type natriuretic peptide; hsPDA, haemodynamically significant patent ductus arteriosus).

In order to set a blood BNP cut-off value for the necessity of surgical ligation, a ROC curve was also drawn using the maximum BNP within the first 5 days of life in Group I: as a result, a BNP cut-off value of 2000 pg/mL was calculated to give the best results: AUC 0.94, TPF 1.0, FPF 0.13 Fig. 4. It is of note that four of six subjects with a maximal BNP level >2000 pg/mL within the five-first days since birth required surgical ligation. In contrast, none of the 14 subjects with <2000 pg/mL of maximum BNP required surgical ligation. Thus, when the cut-off value of the BNP for the necessity of a surgical ligation for hsPDA was set to 2000 pg/mL, it generated an odds ratio as high as 52.2 (95% CI, 2.1 to 1300, p = 0.021); surgical ligation rate was 66.7%.

## Discussion

In the current study, we explored whether the early postnatal blood BNP level could predict the necessity of therapeutic intervention for hsPDA in premature infants. The results are summarized as follows: (i) In the early neonatal period, the blood BNP level was significantly higher in premature infants with hsPDA than in those without hsPDA during postnatal 24–96 h; (ii) Premature infants with hsPDA demonstrated a considerable variations in blood BNP level in early neonatal period at the peak of postnatal 24–48 h, while those without hsPDA did not show such a significant changes; (iii) A cut-off value of 250 pg/mL at postnatal 24–48 h for predicting the necessity of indomethacin treatment for hsPDA by the ROC curve yielded the reasonable odds ratio as high as 5.0. Similarly, a cut-off value of 2000 pg/mL as a maximal BNP level within the first 5 days of life predicting the surgical ligation by the ROC curve also yielded the odds ratio as high as 52.2.

Regarding the timing of indomethacin treatment and the DA closure rate, it was previously reported that early treatment increases the closure rate 4,5. Therefore, we postulate that indomethacin may be considered to be administered if the blood BNP value is above 250 pg/mL in premature infants with hsPDA at the postnatal 24–48 h based on our findings. This approach could allow for the first treatment to be administered a day earlier and could reduce the total amount of indomethacin administered and the number of premature infants with hsPDA who required surgical ligations. All of the subjects who exhibited refractoriness to indomethacin treatment and required a surgical ligation had a maximal BNP level of more than 2000 pg/mL measured within the first 5 days of life. In agreement with our findings, Hsu et al. reported that the indomethacin response is poor in infants with a BNP level of greater than 1805 pg/mL, raising the possibility of a surgical ligation being necessary 14.

It has been reported that the BNP level is higher in infants with congenital heart disease and in premature infants with hsPDA 10–13. Therefore, we consider that the higher the blood BNP level, the more the cardiac stress via DA is and that it can be expected that BNP is a useful biomarker to assess the need for medical intervention such as indomethacin treatment or surgical ligation for hsPDA. In fact, several reports have tried to determine the cut-off value of BNP as a marker for the need for medical intervention in premature infants with hsPDA 14–19. The timing of the closure of DA may explain why the BNP level was increased high in premature infants with hsPDA (Group I), that is, BNP secretion was likely enhanced by ventricular volume overload, which is induced by the left-to-right shunt via a DA 20. In fact, we previously confirmed the significant positive correlation between blood BNP level and the diameter of DA 11.

However, the reported cut-off values for BNP to assess hsPDA varied considerably. This might be due to the considerable differences in the assay method and timing of the blood sampling. For example, the cut-off value by Kalra et al. was lower than that of our study (123 ng/L vs 250 pg/mL) 21. It seems to be due to a difference regarding the timing of blood sampling: that is, the cut-off value was calculated using the data at postnatal 24–48 h in our study, whereas it was carried out by the data obtained at 3–7 postnatal days in the Kalra’s study. The significant negative correlation between BNP levels and the postnatal days of life reported by da Graca may further support our hypothesis 12.

There are several limitations in the study. First, as the study was retrospective in nature, it was not fully blinded. This might influence the therapeutic strategy. Second, measurement of blood BNP using Shionospot BNP® is not global standard in contrast to a widely accepted assay of N-terminal pro-brain natriuretic peptide (NT-proBNP) because of its greater stability and longer half-life 22. This precludes comparing the data between others and ours. Nonetheless, we have chosen BNP over NT-proBNP. This is because it is beneficial for practical use in premature infants with anaemia that Shionospot BNP® requires tiny amount of whole blood as small as 70 μL for measurement in contrast to 1 mL or more for measurement of serum NT-proBNP using an electrochemiluminescence immunoassay at our institution.

In summary, we reached the conclusion that a BNP level more than 250 pg/mL at postnatal 24–48 h and that above 2000 pg/mL within the first 5 days of life indicate the high possibility of indomethacin treatment and surgical ligation, respectively. We believe that measurement of the blood BNP during early neonatal period can be a useful biomarker to assess the hsPDA in premature infants. Future study will clarify to provide a useful biomarker to make decision for therapeutic strategy in those infants.

**Key notes**

- Serial measurements of blood BNP during the first 5 days of life revealed that premature infants with hsPDA demonstrated higher BNP peaking at postnatal 24–48 h than those without hsPDA.
- We deduced the predictive value of 250 and 2000 pg/mL of BNP for indomethacin treatment and surgical ligation, respectively.
- Blood BNP level is useful to assess the necessity of treatment for hsPDA.
